# Additive genetic variance for traits least related to fitness increases with environmental stress in the desert locust, *Schistocerca gregaria*

**DOI:** 10.1002/ece3.8099

**Published:** 2021-09-21

**Authors:** Marie‐Pierre Chapuis, Benjamin Pélissié, Cyril Piou, Floriane Chardonnet, Christine Pagès, Antoine Foucart, Elodie Chapuis, Hélène Jourdan‐Pineau

**Affiliations:** ^1^ CIRAD CBGP Montpellier France; ^2^ CBGP CIRAD Montpellier SupAgro INRA IRD Univ Montpellier Montpellier France; ^3^ Department of Biology University of Nebraska at Kearney Kearney Nebraska USA; ^4^ CIRAD UPR B‐AMR Montpellier France; ^5^ MIVEGEC Université de Montpellier CNRS IRD Montpellier France; ^6^ CIRAD UMR PVBMT Saint‐Pierre France; ^7^ CIRAD UMR ASTRE Montpellier France; ^8^ ASTRE Univ Montpellier CIRAD INRA Montpellier France

**Keywords:** climate change, growth, life history evolution, morphology, pest insects, pigmentation, stress, temperature, viability

## Abstract

Under environmental stress, previously hidden additive genetic variation can be unmasked and exposed to selection. The amount of hidden variation is expected to be higher for life history traits, which strongly correlate to individual fitness, than for morphological traits, in which fitness effects are more ambiguous. However, no consensual pattern has been recovered yet, and this idea is still debated in the literature. Here, we hypothesize that the classical categorization of traits (i.e., life history and morphology) may fail to capture their proximity to fitness. In the desert locust, *Schistocerca gregaria*, a model organism for the study of insect polyphenism, we quantified changes in additive genetic variation elicited by lifetime thermal stress for ten traits, in which evolutionary significance is known. Irrespective of their category, traits under strong stabilizing selection showed genetic invariance with environmental stress, while traits more loosely associated with fitness showed a marked increase in additive genetic variation in the stressful environment. Furthermore, traits involved in adaptive phenotypic plasticity (growth compensation) showed either no change in additive genetic variance or a change of moderate magnitude across thermal environments. We interpret this mitigated response of plastic traits in the context of integrated evolution to adjust the entire phenotype in heterogeneous environments (i.e., adaptiveness of initial plasticity, compromise of phenotypic compensation with stress, and shared developmental pathway). Altogether, our results indicate, in agreement with theoretical expectations, that environmental stress can increase available additive genetic variance in some desert locust traits, but those closely linked to fitness are largely unaffected. Our study also highlights the importance of assessing the proximity to fitness of a trait on a case‐by‐case basis and in an ecologically relevant context, as well as considering the processes of canalization and plasticity, involved in the control of phenotypic variation.

## Graphical abstract

The idea that environmental stress can unmask cryptic additive genetic variation and expose it to selection is well accepted. Still, no consensus has been reached on the potential magnitude of these stress‐induced changes. In the desert locust (*Schistocerca gregaria*), we show that ecologically relevant environmental stress increases available additive genetic variance only for traits that are not directly linked to individual fitness. Our results suggest that using a life history versus morphology categorization of traits as a proxy for their link to fitness might be too simplistic, and instead, traits' proximity to fitness should be known specifically in the studied populations. Our study also highlights that traits' control of phenotypic variation (canalization vs. plasticity) should not be overlooked.

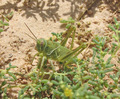

## 1 INTRODUCTION

Global change increases the frequency and variability of stressful climatic events, which can drive dramatic changes in natural population dynamics, such as extinctions in endangered populations (Parmesan, 2006) and invasions in pest populations (Boggs, 2016; Pareek et al., 2017). Besides range shift in pursuit of more favorable environmental conditions (Travis et al., 2013), environmental stress in nature can be buffered in situ, in the short term by phenotypic plasticity (e.g., in behavioral, morphological, and/or physiological traits; Atkinson & Sibly, 1997) and/or, at a longer time scale, by adaptive evolution. Adaptation of populations to a stressful environment is contingent on their overall level of additive genetic variation (*V*_A_) for fitness (Fisher, 1930). While it is well known that *V*_A_ of traits may vary across environments in natural populations (Hoffmann & Parsons, 1991; Wood & Brodie, 2015), there is a lack of consensus in literature on how its magnitude and direction is affected by stress (Charmantier & Garant, 2005; Fischer et al., 2021; Hoffmann & Merilä, 1999; Rowinski & Rogell, 2017).

Various explanations have been proposed to explain the inconsistent effects of environmental stress observed on trait evolutionary response, including the choice of quantitative genetic parameters, the nature of the stressor considered, and the link of the traits to individual fitness in populations under study. A short review on these sources of variation in observed evolutionary responses to stress is provided in Information Box 1. Here, we reconsider the universality of the broadly accepted assumption that life history traits suffer stronger purging selection, because of a tighter link to individual fitness than morphological traits (Visscher et al., 2008) and thereby the validity of the trait classification (life history vs. morphology) usually employed in stress evolution studies to derive theoretical expectations. We rather argue that the link of traits to individual fitness might need to be evaluated with caution on a case‐by‐case basis, in particular, since it depends significantly on the type of the organism studied and may vary in different ecological settings. For example, in ectotherms, morphological traits related to body size are strongly correlated with growth and fecundity, two crucial components of fitness (Mousseau & Roff, 1987). Similarly, while life history traits are usually tightly linked to fitness, their selection regime may constrain or else preserve their amount of genetic variation. For example, while some fitness components are under strong stabilizing selection that canalizes a single optimal phenotype across the natural range of environments and lessens additive genetic variance, others are under antagonistic selection across natural environments or balancing selection that drives several optimal phenotypes and significant additive genetic variance.

BOX 1 **Sources of variation in observed evolutionary responses to stress**

- Quantitative genetic estimates

Conclusions about the effect of stress on traits' potential to respond to selection can be affected by the usual focus in literature on trait heritability (*h* ^2^), the fraction of the total phenotypic variation due to additive genetic effects (*V*_A_). However, this measure scales *V*_A_ over the residual nongenetic variance components (*V*_R_) that may be sensitive to environment (Houle, 1992), at least for some life history traits that display developmental instability under certain stress conditions (Charmantier & Garant, 2005; Rowinski & Rogell, 2017). In these cases, an increase in environmental variation with stress would result in a mechanical decrease in *h* ^2^, without *V*_A_ even being changed, questioning the appropriateness of *h* ^2^ as an estimate of traits' evolutionary potential (Houle, 1992). To mitigate these misunderstandings, studies should report variance components of heritability, if not use alternative measures, such as evolvability, that scale *V*_A_ over the phenotypic mean rather than *V*_P_ (Hansen et al., 2011; Houle, 1992).

- Nature of the stressors

Another explanation for the lack of a consensus comes from the very nature of the stressor(s) studied. Under global changes, most environmental alterations are expected to be gradual, sustained changes in the frequency of environmental experiences. However, most studies focus on either extreme, lethal stresses on resistance traits (e.g., heat knockdown or chill coma recovery, Hoffmann & Parsons, 1991; Imasheva et al., 1998; Sgro & Hoffmann, 1998; Sisodia & Singh, 2009) or sublethal environments not previously experienced (e.g., outside the geographical or seasonal populations' normal ranges; Husby et al., 2011; McGuigan et al., 2011). The later studies tend to confound adversity with novelty of the environment (Charmantier & Garant, 2005; Schlichting, 2008), even though the assumption that environmental novelty affects traits' additive genetic variance has recently been challenged by Wood and Brodie (2015) and Rowinski and Rogell's (2017) meta‐analyses. The timescale populations experience the stress also conditions its effects. When induced in a single generation, stress effects are limited to environment‐specific allelic effects, often modulated by variance in gene expression in complex gene regulatory networks (Bergman & Siegal, 2003; Frankel et al., 2010). When accumulated on a longer timescale, stress effects can also result from evolutionary changes in the genetic architecture (e.g., selection, mutation, or recombination that shapes allelic frequencies or combinations).

- Link of the traits to individual fitness

Meta‐analysis studies on animals have mainly investigated two categories of traits: life history traits (e.g., reproductive output, survival, and developmental performance), and morphological traits (e.g., body pigmentation and body shape). The rationale behind this comparison is that the level of connection of a trait to individual fitness may determine its level of additive genetic variation (including hidden variation) because purging selection is assumed to be stronger on traits that are tightly linked to fitness (Mousseau & Roff, 1987). There is evidence that *V*_A_ of life history traits, presumably closely linked to fitness, tends to be insensitive to the level of environmental stress experienced by the population (see Charmantier and Garant's (2005) meta‐analysis, but also Saxon et al., 2018, and Fischer et al., 2021). In contrast, an increase in *V*_A_ with environmental stress has been ascertained for morphological traits, less expected to contribute to fitness (see Hoffmann and Merilä's (1999) meta‐analysis, but also De Moed et al., 1997). However, this trend for a stress‐dependent increase in *V*_A_ that mainly stands for morphological traits has been recused by Rowinski and Rogell's (2017) meta‐analysis, which showed a general increase in *V*_A_ with environmental stress, irrespective of the category of the traits considered.

Furthermore, some traits are under more indirect selection that favors adaptive phenotypic plasticity to buffer the fitness effects of environmental instability. By reducing selection pressures on fitness components, adaptive phenotypic plasticity is expected to hide genetic variation from natural selection. This hidden variation can be unmasked under an environmental stress that increases the strength of selection on fitness components enough to make adaptive phenotypic compensation incomplete (Paaby & Rockman, 2014; Schlichting, 2008). Although this theoretical hypothesis makes the prediction that the magnitude of change in *V*_A_ should correlate with the difference in phenotypic optima between environments, it has not been validated by previous research (Wood & Brodie, 2015). On the one hand, the lack of support for this correlation may result from the confounded analysis of traits showing both adaptive and nonadaptive phenotypic plasticity. Indeed, unlike for traits with adaptive plasticity, new environments induce phenotypes further away from the new local optimum for traits with nonadaptive plasticity, which results in a reduction in individual fitness and predicts a strong directional selection (Ghalambor et al., 2007, 2015). On the other hand, the predicted increase in *V*_A_ for traits exhibiting adaptive plasticity might actually be constrained by the genetic variance–covariance matrix in which these traits evolved. This is particularly relevant to pleiotropic genetic correlations among traits sharing the same developmental pathways (Ellers & Liefting, 2015; Paaby & Rockman, 2014; Schlichting, 2008; Snell‐Rood et al., 2010; Stearns, 1992).

In any case, large variation in proximity to fitness between traits of a same category (life history and morphology) likely generates noise in the meta‐analyses' data, blurring the nature of the observed relationship between environmental stress and *V*_A_. By studying many traits for which environmental stress sensitivity and evolutionary significance are known in natural populations, one can circumvent the need to rely on the potentially biased shortcut of using trait category as a proxy of its relationship with individual fitness. To our knowledge, this approach has rarely been undertaken in between‐environment quantitative genetic comparisons (see Table S1 from the Wood and Brodie's (2015) meta‐analysis). Herein, we propose such an approach in the desert locust (*Schistocerca gregaria* (Forskål, 1775)), an agricultural pest and a model organism for the study of insect polyphenism. Crucially, phenotypic integration has been scrutinized in this species for the past century (Simpson et al., 2011; Uvarov, 1921), including plastic potential in stressful environments. Indeed, desert locust populations experience highly variable environmental conditions, in deserts and by virtue of extensive migration, in response to which it can exhibit extreme phenotypic plasticity affecting a large diversity of traits (see Table S1 in Section S1 in the Supporting Information for a literature review of environmental effects on locust phenotypes).

In this study, solitarious locusts from a natural population reared under controlled environmental conditions were subjected during their lifetime to either an optimal warm temperature or a sublethal cold temperature, respecting the range of developmental temperatures experienced in natural habitats, and thereby avoiding the pitfall of both novelty and extreme severity of environments. We investigated ten traits related to life history and morphology, and for which literature evidence exists on their presumed relationship with fitness. Previous knowledge on their recognized biological functions and selective regimes, and on their observed phenotypic variability, phenotypic plasticity, and genetic variance under benign environment is given in Information Box 2. We hypothesized that, under thermal stress, *V*_A_ would be unaffected for traits strongly and directly related to fitness (body weight at adult emergence, nymphal viability, and the allometry of the fore wing involved in dispersal), while it would be increased in traits whose link to fitness is weak (background pigmentation and allometries of the head and of the eye) or indirect presumably through adaptive phenotypic plasticity (intra‐instar growth rate, occurrence of extramolting, development time, and melanin pigmentation). However, expected change for those latter plastic traits is not easy to quantify, though it might be rather low on average, with responses likely to be more specific, depending both on the intensity at which phenotypic compensation is compromised with stress and on the genetic constraints in which they evolve. Beyond evaluating *V*_A_ predictions in relation to environmental stress in a desert locust population, thereby expanding the range of taxa for which values of genetic parameters have been obtained, our results should help to predict more accurately the future range and severity of attacks by this agricultural pest in response to global change over long timescales (Ayali, 2019; Maeno et al., 2021; Meynard et al., 2017, 2020).

BOX 2 **Literature‐based evidence for evolutionary significance of the 10 traits measured in this study**

- Traits related to life history

We investigated five traits involved in development time, nymphal viability, and adult body size. The body weight at adult emergence is a crucial trait closely related to individual fitness, optimized by natural selection for offspring production and for starvation resistance in this species experiencing low‐quality environments and costly long‐distance migration (Gotthard, 2001; Injeyan & Tobe, 1981). This trait shows relatively limited phenotypic variability, including with environment, and null genetic parameters, including heritability and evolvability, under benign environment (Maeno & Tanaka, 2008; Pélissié et al., 2016). Such a lack of variability is expected for a trait genetically canalized under strong stabilizing selection (Pélissié et al., 2016). Strong phenotypic plasticity during juvenile development allows to adjust for adult body size in order to compensate for small hatchling size and/or low intra‐instar growth rate in adverse conditions (Esperk et al., 2007; Honěk, 1993; Maeno & Tanaka, 2008; Nijhout, 1975; é et al., 2016; Stearns, 1992). In *S. gregaria*, the plasticity exhibited by the probability of extramolting and the development time under stressful environments is likely adaptive, with phenotypes altered in the direction that allows the minimum body size for successful reproduction to be reached. In contrast, stronger physiological constraints within the successive instars are enough to explain plastic responses of the overall growth rate under low‐quality environments (Esperk et al., 2007; Nijhout, 1975). Finally, a high level of genetic variance was observed for developmental traits involved in adjustment of body size (i.e., intra‐instar growth rate, occurrence of extramolting, development time), which can partly be explained by antagonistic selection between heterogeneous environments (e.g., seasons; Pélissié et al., 2016).

- Traits related to morphology

We investigated five traits involved in body pigmentation and body shape. Most of these morphological traits exhibit plasticity, primarily in response to local population density (but not only, see, e.g., Table S1 in Section S1 in the Supporting Information). When juvenile individuals in recently dense populations transition from a solitarious to a gregarious phenotype, their pigmentation traits are particularly affected. Solitarious late juveniles are typically green, while gregarious late juveniles display a beige or yellow background color with dark melanin patterns. Adult desert locusts in their gregarious phase tend to develop larger heads, longer wings, and smaller eyes than their solitarious counterparts. The ratio of the length of the hind femur on the maximum width of the head is the most phase‐discriminating morphometric variable in *S. gregaria* (Dirsh, 1953). Although the morphological traits we chose are primarily associated with density‐dependent phase phenotypes, two of them are also involved in functions explicitly affecting fitness, in a broad range of environments. Melanin pigmentation is an important component of insect cuticle and allows insects to increase their absorption of solar radiations, heat up faster, attain higher body temperatures, and be more resistant to pathogens (Fedorka et al., 2013; Majerus, 1998; May, 1979). This trait generally exhibits a high level of phenotypic variability in response to both lifetime and parental environments (see Table S1 in Section S1 in the Supporting Information). Furthermore, the production of melanin is known to be costly and to be in trade‐off with other life history processes requiring significant energy input (see also Prokkola et al., 2013; Roff & Fairbairn, 2013), suggesting that plastic responses may become maladaptive under certain environments. Allometric wing length is correlated to performance of dispersal flight (Berwaerts et al., 2002), which is under strong selection pressure in this migratory species that must face deserts' extreme environmental fluctuations (Roffey & Magor, 2003). Indeed, while gregarious populations migrate in spectacular swarms comprising millions of individuals, solitarious populations also migrate across long distances that follow intertropical rain fronts, mostly during the night (Chapuis et al., 2014; Roffey & Magor, 2003).

## 2 MATERIALS AND METHODS

### 2.1 Study population

Our laboratory desert locust population was derived from 57 fertilized wild females collected in Mauritania in December 2010 (see Pélissié et al., 2016, for further details). The parentage relationships of the emerging offspring (G1) was inferred based on multilocus genotyping as described in Pélissié et al. (2016). We then standardized rearing for three successive generations (G2 to G4) prior to phenotypic measurements (G5): Locusts were maintained under isolated conditions in 1‐L individual plastic cages, at 34.0℃ and 55% humidity, under a photoperiod of 12/12 (L:D) hours, and fed ad libitum with fresh wheat shoots and bran. Three generations of rearing in a common environment controlled for shared transgenerational environment effects (e.g., nonheritable maternal effects) that might contribute to sibling resemblance so that the remaining parental variance could be attributed to genetic variation among parents, (uncontrolled) micro‐environmental variation, and gene‐by‐environment interactions, rather than parental environment (see Section S1 in the Supporting Information for further details on procedures for minimizing parental environmental effects in our experiment and a literature review of the effects of parental environment on locust phenotypes). To prevent the loss of genetic diversity due to drift and inbreeding, we maintained a population size of several hundreds of individuals using discrete generations and mated isolated males and females originating from different parents. As a result, genetic variation among the individuals within the generation of *V*_A_ measurement was substantial, as measured by the expected heterozygosity at 19 neutral microsatellite loci (0.63 ≤ *H*_E_ ≤ 0.92; results not shown).

### 2.2 Thermal stress

The temperature treatments, applied at G5, were chosen to reflect sublethal seasonal thermal stress. Indeed, winter lower temperatures are particularly challenging for the desert locust adapted to desert's harsh heat. For example, juveniles are usually not able to develop to the adult stage under temperature below 24℃ (Husain & Ahmad, 1936) and large variation in hatchling‐to‐adult development time between summer and winter seasons has been observed in nature (i.e., range of 24–57 days in the solitarious phase; Symmons et al., 1974; Wardhaugh et al., 1969). Growth temperatures were 34℃ and 26.5℃ on average and within the normal viable range of the species: For example, at the source population's geographical coordinates, mean temperatures are 33.2℃ and 22.9℃ for the warmest and coldest trimesters (www.worldclim.org). The temperature treatments included some daily variation, with colder nights than days (95% confidence intervals = 32–36℃ and 25–28℃, respectively). The warm treatment (34℃) was chosen to optimize nymphal growth. The cold treatment (26.5℃) was set to stress it, while limiting strong negative effects on survival (Dudley, 1961; Hamilton, 1936, 1950; Hoste et al., 2002).

### 2.3 Experimental design

For each trait, we estimated the additive genetic component of phenotypic variance based on a half‐sib/full‐sib design, which is not biased by dominance variance, allowing for the estimation of narrow‐sense heritability (*h* ^2^) (Lynch & Walsh, 1998). We applied a paternal crossing scheme (at G4), where eight males were successfully mated with one to three virgin females, leading to a total of 15 maternal families (i.e., two, five, and one males mated to one, two, and three females, respectively). For each maternal family, 5–56 G5 offspring were evenly distributed (within 12 h after hatching) between warm and cold treatments, for the entire duration of their nymphal development (until death or adult molt). Individual positions were randomized weekly within the climatic chambers to control for potential environmental microvariation. Nymphal mortality in the G5 was 46% (in line with most other laboratory studies of the desert locust; e.g., Hoste et al., 2002) and reduced the final sample size to 256 adults. We constructed a 5‐generation pedigree matrix to be used in quantitative genetic analyses (see Section S2 in the Supporting Information).

### 2.4 Nymphal life history traits

We monitored the development of all G5 individuals from hatching day to adult emergence. Each day, we weighted the nymphs to the nearest milligram and recorded any molting event. *Growth Rate* was calculated within each nymphal instar as the slope coefficient of the log‐linear regression of body weight on time from last molting event to the day at maximal instar weight (Pélissié et al., 2016). In this species, individuals compensate for lower growth rate by undergoing an extramolt, between the third and fourth instar. We confirmed the occurrence of extramolting by assessing the number of dark stripes on late‐instars' eye, which correlates with the number of nymphal instars (Mukerji & Batra, 1938). *Extramolting* was recorded as a binary indicator: 0 = 5 instars (normal) or 1 = 6 instars. *Development Time* was the age (in days) at which an individual reaches its adult emergence. *Maximal Nymphal Weight* was the maximal weight attained during the last instar. *Nymphal Viability* was the probability of hatchlings to reach adulthood recorded as a binary indicator: 0 = failure or 1 = success.

### 2.5 Morphological phase traits

We considered two commonly studied sets of morphological phase traits: fifth‐instar nymphal pigmentation and adult morphometry (Pener & Simpson, 2009). Color differences between gregarious and solitarious nymphs are the most noticeable phase changes (Nickerson, 1956). We measured the percentage of dark color (*Dark Pigmentation*) and the percentage of green color (*Green Pigmentation*), which are estimates of the melanin and background polyphenisms of an individual (see Section S3.1 in the Supporting Information for details on methods and illustrations). We measured three morphometric ratios that encapsulate morphometric changes: *Length of the fore wing/Length of the hind femur* and *Length of the hind femur/Maximum width of the head*, used for phase state characterization in the field (Stower et al., 1960), and *Vertical diameter of eyes/Width of the vertex between eyes*, considered as a reliable indicator of phase change (Dirsh, 1953) (see Section S3.2 in the Supporting Information for details on methods and illustrations).

### 2.6 Statistical analyses

We first analyzed each of the 10 traits using linear mixed models in R 3.2.3 (R Core Team, 2017) using the ***stats*** package, testing for the effects of *Temperature*, *Sex*, *Extramolting, Hatching Weight*, and all interactions between pairs of factors. We performed a backward stepwise model selection using the Akaike information criterion (AIC) and tested the significance of factors present in the final model with an ANOVA (type III) using the ***MASS*** package. We then ran the quantitative genetic analyses, using ***ASReml‐R*** (Gilmour et al., 2009), within each temperature treatment separately. For each trait, we estimated the additive genetic component of phenotypic variance by running an animal model that specifies a genetic effect as a random pedigree effect: *Y_i_* = *µ* + *A_i_* + *ε_i_*, where *Y_i_* is the phenotype of individual *i*, *µ* is the population phenotypic mean, *A_i_* is the individual's additive genetic value, and *ε_i_* is the random residual value. The total phenotypic variance (*V*_P_) was then decomposed into additive genetic (*V*_A_) and residual (*V*_R_) components, such that *V*_P_ = *V*_A_ + *V*_R_. The relatedness matrix was based on a pedigree spanning 5 generations, which incorporated not only data from the full‐sib and half‐sib relatives of the 4th‐generation families, but also data from genealogical relatedness since the field capture of individuals (see Section 2.3 in the main text and Section S2 in the Supporting Information). We tested for significant *V*_A_ using a likelihood‐ratio test (LRT) against a null model. We report observed *p*‐values and their Shannon information transforms *S*‐value = −log_2_(*p*‐value), which measures the degree of incompatibility between the data and the null hypothesis of the model (Greenland, 2019; Wasserstein et al., 2019). The *S*‐value rounded up to the nearest whole number is the number of units of binary digits (bits) of information against the null hypothesis of the model. Since heritability estimates tend to increase with the number of fixed effects in the model (de Villemereuil et al., 2018; Wilson, 2008), and in order to obtain conservative estimates, we ran animal models on raw phenotypic data (i.e., without including the effects of *Sex*, *Extramolting*, or *Hatching Weight*). Note that using mixed‐effects models including covariates did not lead to any significant change in *V*_A_ or *h* ^2^ estimates (results not shown). Only *Growth Rate* was analyzed including nymphal stage as a covariate and individual identity as random effect, because of the need to account for the longitudinal structure of the dataset (Wilson et al., 2010). We estimated narrow‐sense heritability as *h* ^2^ = *V*_A_/*V*_P_, except for binary traits *Extramolting* and *Nymphal Viabilit*y, for which *h* ^2^ = *V*_A_/(*V*_R_ + *V*_A_ + 1) with *V*_R_ = 1 (Nakagawa & Schielzeth, 2010; de Villemereuil, 2018). For these two traits, we used a generalized linear mixed model with a binomial distribution and a probit link function, equivalent to a threshold model (Nakagawa & Schielzeth, 2010; de Villemereuil, 2018). We also derived a measure of evolvability, as *I*_A_ = *V*_A_/*μ* ^2^, with *μ* the phenotypic mean, which is more robust to the presence of fixed effects in animal models (Hansen et al., 2011). *I*_A_ can directly be interpreted as the expected percent change in a trait under a unit strength of selection (Hansen et al., 2011), but it is inappropriate for binary traits, for which *V*_A_ is estimated on a latent (liability) scale (de Villemereuil, 2018). Standard errors for *V*_A_, *V*_R_, *h* ^2^, and *I*_A_ were obtained using the delta method (Garcia‐Gonzalez et al., 2012; Lynch & Walsh, 1998).

### 2.7 Simulation‐based power analyses

Under the typically limited sample size of our study, we were concerned by a low statistical power of our quantitative genetic analyses. Thus, we performed simulations based on our experimental design at the low temperature treatment, using our pedigree and the smallest and largest sample sizes present in our dataset (here *N* = 108 for the morphometric ratio *Vertical diameter of eyes/Width of the vertex between eyes* and *N* = 243 for the *Nymphal Viability*). We simulated individual phenotypic value of the *i*th individual such that *Y_i_* = *A_i_* + *ε_i_*. *A_i_* was the breeding value of the individual *i* and was normally distributed assuming *V*[*A_i_*] = *V*_A_ (*A* is the additive relationship matrix computed based on the pedigree). *ε_i_* was the random residual variation and was normally distributed with variance *V*_R_ (the mean phenotype in the population was arbitrarily set to 0; Morrissey et al., 2007). To simulate breeding values *A_i_*, we used the ***rbv*** function from the R package ***MCMCglmm*** (Hadfield, 2010). To investigate different scenarios, we manipulated *V*_R_ so that *h* ^2^ estimates increased from 0 to 1. For each level of heritability, we simulated 1,000 phenotypic datasets. Finally, though our experiment was designed to minimize maternal effects, we considered their potential influence on our analyses. To this aim, we also assumed a variance *V*_M_ = 0.1 and simulated individual phenotypic values such that *Y_ik_* = *A_i_* + *M_k_* + *ε_jk_*, where *M_k_* was the normally distributed maternal effect.

## 3 RESULTS

### 3.1 Phenotypic differences between temperature treatments

**FIGURE 1:**
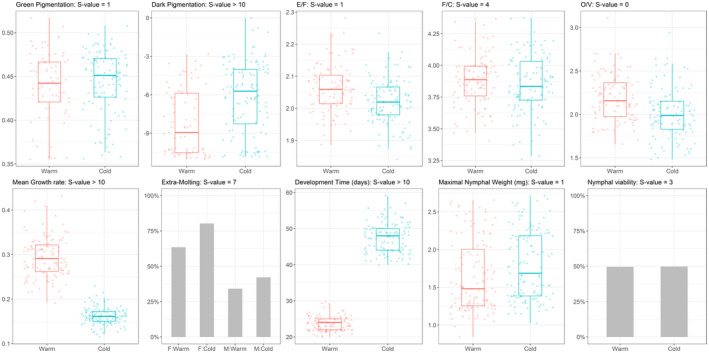
Boxplot or proportional representations of morphological and nymphal life history traits measured in the desert locust under the warm and cold environments. In boxplots, point values are also represented, in a triangular shape for males (M) and in a round shape for females (F). Extramolting data are represented for each environment by sex group. Nymphal viability could not be represented by sex since nymphs that died early in their development have not been sexed. Other traits than maximal nymphal weight and development time are dimensionless (i.e., binary, proportions, or ratios). Dark pixel data were logit‐transformed (see Section S3.1 in the Supporting Information for further details). For the sake of clarity, warm and cold environments are depicted in red and blue colors, respectively. See Table S3 in Section S4 in the Supporting Information for details on tests for statistical values

Figure 1 depicts the phenotypic distributions of the 10 traits studied under optimal or stressful temperatures. Section S4 in the Supporting Information presents sample sizes, means, standard deviations, and statistical values observed from an analysis of variance on model selection. Cold treatment triggered a plastic response for traits involved in developmental plasticity for adjustment of body weight, but not for nymphal viability and body weight. In average, development time was twice longer and growth rate was twice lower under low temperature than under high temperature. Nymphs' probability to undergo an extramolt was increased by 25% (i.e., 34% vs. 42% in males, and 63% vs. 80% in females for high vs. low temperature). Although morphological traits were skewed toward darker individuals and males with larger heads under low temperature, these phenotypic effects remained small. For example, most desert locust nymphs remained pale at their last growth stage, with a color pattern typical of the *solitarious* phase (i.e., types A‐B in Hunter‐Jones, 1958), and only four insects showed a substantial amount of melanin typical of the *gregarious* phase (type H in Hunter‐Jones, 1958) or of an intermediate phenotype (types D and E in Hunter‐Jones, 1958). Finally, melanic pigmentation was the single morphological trait in our study to be significantly associated with values of key life history traits of low quality, namely long development time and low size at adult molt (Section S5 in the Supporting Information). These results are in agreement with previous findings on temperature effects on color, morphology, and development of juvenile locusts (see Section S1 in the Supporting Information for a literature review).

### 3.2 Genetic differences between temperature treatments

The observed effects of the sublethal cold stress on genetic parameters (i.e., raw additive genetic variance and its standardized measures, heritability, and evolvability) varied between the 10 traits studied in the desert locust population (see Tables 1 and 2 for optimal and stressful temperatures, respectively).

**TABLE 1:** Estimates of genetic parameters for the 10 traits studied in the desert locust from the subsets of individuals reared under warm environment

*Trait*	*N*	*μ*	*SE*(*μ*)	*h* ^2^	*SE*(*h* ^2^)	*I*_A_	*SE*(*I*_A_)	*p*‐value	*S*‐value
Morphological phase traits									
Green Pigmentation	76	0.440	4.36E−03	0.396	3.04E−01	0.323%	1.02E−01	1.74E−01	2.520
Dark Pigmentation	77	0.004	1.01E−03	0.155	3.47E−01	NA	NA	3.11E−01	1.683
Length of the fore wing/Length of the hind femur	104	2.061	7.13E−03	0.104	1.61E−01	0.013%	1.64E−03	4.91E−01	1.026
Length of the hind femur/Maximum width of the head	107	3.885	1.94E−02	0.021	1.29E−01	0.006%	7.46E−04	9.05E−01	0.144
Vertical diameter of eyes/Width of the vertex between eyes	105	2.190	2.61E−02	0.000	1.00E−09	<0.000%	4.96E−03	1.00E+00	0.000
Life history traits									
Growth Rate	119	0.293	4.24E−03	0.058	2.94E−02	0.751%	3.38E−01	8.19E−04	10.254
Extramolting	141	0.475	4.22E−02	0.322	1.40E−01	NA	NA	8.21E−04	10.251
Development Time	119	23.681	1.78E−01	0.290	3.57E−01	0.206%	1.74E−03	8.50E−02	3.556
Maximal Nymphal Weight	130	1.633	4.13E−02	0.088	1.20E−01	0.734%	2.15E−02	3.10E−01	1.690
Nymphal Viability	240	0.496	3.23E−02	0.263	1.18E−01	NA	NA	1.87E−04	12.382

Note

We presented sample sizes (*N*), values for phenotypic mean (*μ*), narrow‐sense heritability (*h* ^2^) and evolvability (*I*_A_, expressed in %), and their standard errors (*SE*), as well as *p*‐ and *S*‐values for the model with the additive genetic variance. Note that for the binary traits, Extramolting and Nymphal Viability, *V*_A_ and *h* ^2^ are computed on a latent (liability) scale and scaling by the data mean (i.e., *I*_A_) is not meaningful. Similarly, scaling by the data mean is not meaningful for the proportional trait Dark Pigmentation that was logit‐transformed. Note that the mean phenotypic value for this trait was here reported from raw (nontransformed) data.

**TABLE 2:** Estimates of genetic parameters for the 10 traits studied in the desert locust from the subsets of individuals reared under cold environment

*Trait*	*N*	*μ*	*SE*(*μ*)	*h* ^2^	*SE*(*h* ^2^)	*I*_A_	*SE*(*I*_A_)	*p*‐value	*S*‐value
Morphological phase traits									
Green Pigmentation	116	0.444	3.31E−03	0.957	2.64E−01	0.804%	7.58E−02	6.47E−07	20.560
Dark Pigmentation	118	0.023	5.88E−03	0.066	1.79E−01	NA	NA	5.40E−01	0.889
Length of the fore wing/Length of the hind femur	109	2.021	6.68E−03	0.029	1.11E−01	0.003%	1.63E−03	7.77E−01	0.365
Length of the hind femur/Maximum width of the head	111	3.853	2.12E−02	0.350	2.29E−01	0.126%	1.21E−03	3.76E−02	4.732
Vertical diameter of eyes/Width of the vertex between eyes	108	2.009	2.76E−02	0.135	1.42E−01	0.276%	7.45E−03	1.12E−01	3.154
Life history traits									
Growth Rate	121	0.162	1.73E−03	0.039	2.72E−02	0.284%	8.21E−01	3.97E−02	4.655
Extramolting	134	0.619	4.21E−02	0.408	1.30E−01	NA	NA	9.99E−05	13.290
Development Time	121	47.570	3.73E−01	0.397	9.80E−01	0.315%	2.14E−03	5.92E−03	7.401
Maximal Nymphal Weight	125	1.792	4.07E−02	0.115	1.46E−01	0.755%	1.72E−02	3.59E−01	1.477
Nymphal Viability	243	0.498	3.21E−02	0.273	1.11E−01	NA	NA	1.59E−05	15.938

Note

We presented sample sizes (*N*), values for phenotypic mean (*μ*), narrow‐sense heritability (*h* ^2^) and evolvability (*I*_A_, expressed in %), and their standard errors (*SE*), as well as *p*‐ and *S*‐values for the model with the additive genetic variance. Note that for the binary traits Extramolting and Nymphal Viability, *V*_A_ and *h* ^2^ are computed on a latent (liability) scale and scaling by the data mean (i.e., *I*_A_) is not meaningful. Similarly, scaling by the data mean is not meaningful for the proportional trait Dark Pigmentation that was logit‐transformed. Note that the mean phenotypic value for this trait was here reported from raw (nontransformed) data.

#### 3.2.1 Nymphal life history traits

The model including a *V*_A_ effect was supported for both temperature treatments in most nymphal life history traits: *Growth Rate*, *Extramolting*, *Development Time*, and *Nymphal Viability* (*S*‐value >4). Narrow‐sense heritability (*h* ^2^) was substantial for *Nymphal Viability*, *Development Time* and *Extramolting* (i.e., 0.26–0.41), but very low for *Growth Rate* (i.e., 0.04–0.06). In contrast, the model including a *V*_A_ effect was not well supported for *Maximal Nymphal Weight* in both temperature treatments (with *S*‐values <2), suggesting that this trait did not display any heritable variation. Evolvability estimates for nymphal life history traits (when computable) were above 0.2%, sometimes reaching nearly one percent. Even body weight showed high evolvability in both optimal and stressful environments (i.e., ~0.75%), in spite of low heritability. These values agree with most studies in insects (Hammerschmidt et al., 2012) and the meta‐analyses of Houle (1992) and Hansen et al. (2011) for this category of traits, which tend to have a high potential for rapid evolution.

#### 3.2.2 Morphological phase traits

The model including a *V*_A_ effect was not supported under an optimal (warm) temperature for most morphological traits (*S*‐values ≤2). This resulted in low heritability (i.e., *h* ^2^ = 0–0.15) and low evolvability (i.e., I_A_ = 0%–0.013%) in the optimal environment. The single exception was *Green Pigmentation*, a proxy of the background pigmentation, with indication of some additive genetic variance (*S‐value = 3*) and quite elevated *h* ^2^ (0.396) and *I*_A_ (0.323%) values. Under the stressful low temperature, the heritability and evolvability values for *Green Pigmentation* more than doubled, with *h* ^2^ reaching 0.957 (the highest value recovered for any trait in our experiment) and a *S*‐value showing an eightfold increase. The two morphometric ratios, *Length of the hind femur/Maximum width of the head* and *Vertical diameter of eyes/Width of the vertex between eyes*, which had nearly null values in the optimal treatment, showed the largest changes in standardized genetic parameters with the sublethal treatment: Their heritability reached 0.35 and 0.135, and evolvability reached 0.126% and 0.276%, respectively, with *S*‐values reasonably supporting models including a *V*_A_ effect (5 and 3, respectively). In contrast, the proxy of melanin pigmentation, and the ratio *Length of the fore wing/Length of the hind femur* still showed negligible raw and standardized genetic parameters in the cold environment, with null *S*‐values.

#### 3.2.3 Overall picture

**FIGURE 2:**
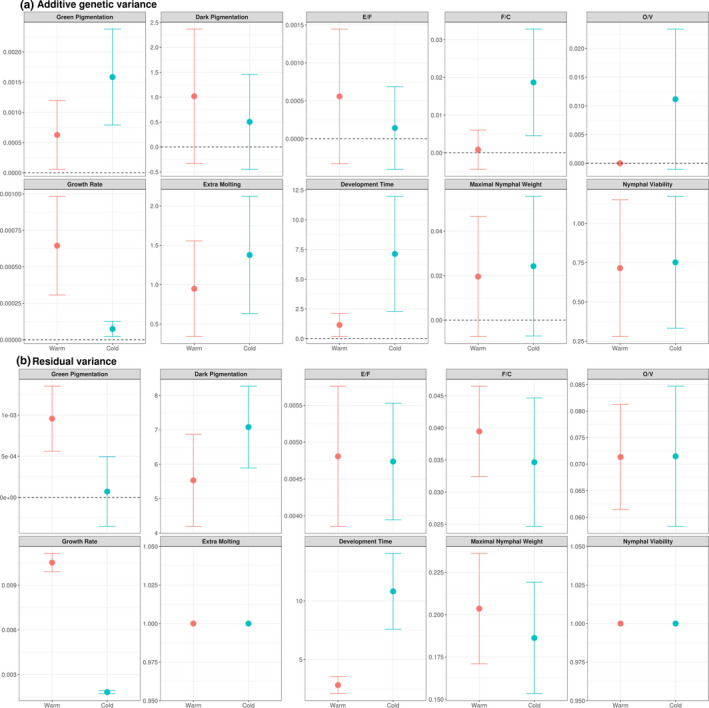
Additive genetic (a) and residual (b) variances of morphological and nymphal life history traits of the desert locust estimated under the warm and cold environments. The compatibility interval is represented by ±the standard error around the estimate. For the sake of clarity, warm and cold environments are depicted in red and blue colors, respectively. No overlap between compatibility intervals of the warm and cold environments can be interpreted as a significant difference in variance. For the binary traits *Extramolting* and *Nymphal Viability*, *V*_A_ is computed on a latent (liability) scale in which *V*_R_ fixed to 1 (de Villemereuil, 2018). Raw data for the proportional trait *Dark Pigmentation* are logit‐transformed to provide a better approximation to the Gaussian distribution. See Section “Material and Methods” for details on computations

Overall, heritability (*h* ^2^) remained at similar level between temperature treatments not only for life history traits but also for the morphological traits related to dispersal and melanism (Table 3). For most of these traits (*Extramolting, Nymphal Viability*, *Maximal Nymphal Weight*, *Dark Pigmentation*, and *Length of the fore wing/Length of the hind femur*), this was due to constant *V*_A_ and *V*_R_ values and phenotypic means across optimal and stressful temperatures (Figure 2). For *Development Time* and *Growth Rate*, a change in *V*_A_ between temperatures (increase and decrease in the cold treatment, respectively), was almost fully compensated by a change in similar direction in phenotypic mean (and *V*_R_) (Figure 2; see also Table 3 for resulting natural logarithmic changes in *I*_A_). In contrast, the three other morphological traits showed marked increases in both standardized levels of additive genetic variance under the cold treatment (Tables 1, 2, 3). For the nymphal green pigmentation, this change was due to both an increase in *V*_A_ and a decrease in *V*_R_ (Figure 2). For the ratios *Femur Length*/*Head Width* and *Vertical diameter of eyes/Width of the vertex between eyes*, the change was entirely due to a 10‐fold increase in *V*_A_ (*V*_R_ remaining constant; Figure 2). Note that these two shape traits were not significantly correlated (Section S5 in the Supporting Information).

**TABLE 3:** Overview of the observed effects of the sublethal cold stress on evolvability for the 10 traits studied

Presumed link to fitness^b^	*Trait*	Main recognized function	Change with stress	
Weak and/or environment‐specific selection	Green Pigmentation	Predation resistance by homochromy^1−3^	.	+ (91%)
	Length of the hind femur/Maximum width of the head	Interindividual interactions^6,7^	.	+ (312%)
	Vertical diameter of eyes/Width of the vertex between eyes	Interindividual interactions^6,7^	.	+ (1,442%)
Environmentally antagonistic selection (growth compensation)	Dark Pigmentation	Thermoregulation, disease resistance^4,5^	+ (179%)	.
	Growth Rate	Adjustment of body size^8^	− (60%)	− (97%)
	Extramolting	Adjustment of body size^8^	+ (27%)	.
	Development Time	Adjustment of body size^8^	+ (70%)	+ (42%)
Strong and direct stabilizing selection	Length of the fore wing/Length of the hind femur	Dispersal ability^a^	.	.
	Maximal Nymphal Weight	Investment into reproduction^9^	.	.
	Nymphal Viability	Viability	.	.

Note

**FIGURE 3:**
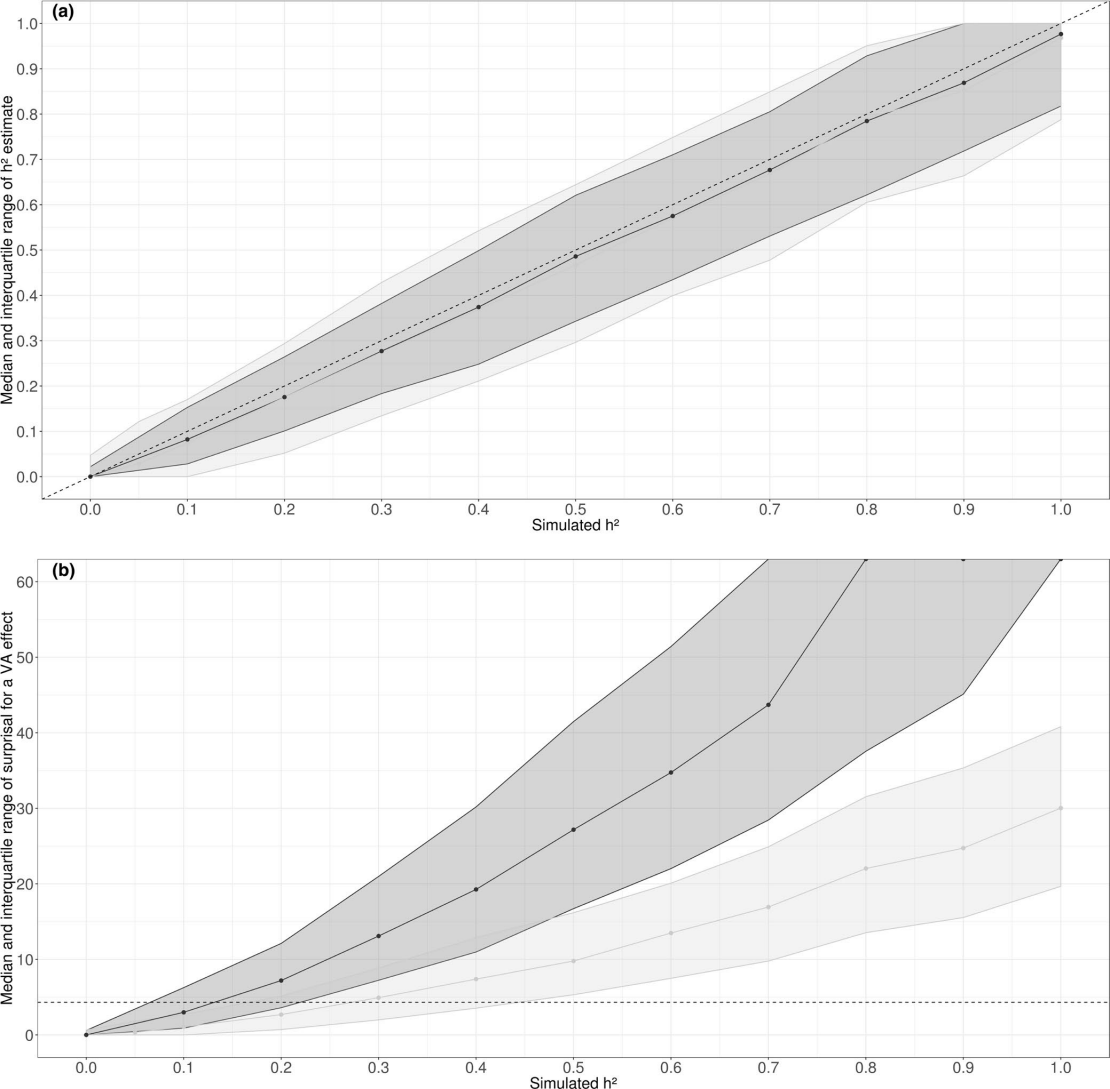
Simulation analysis of the statistical power of our quantitative genetic analysis. We set 11 levels of heritability (i.e., 0. 0, 0.1, 0.2, 0.3, 0.4, 0.5, 0.6, 0.7, 0.8, 0.9, and 1.0), and, for each level, we simulated 1,000 phenotypic datasets based on our experimental design at the low temperature, that is, with exactly the same pedigree as for the subset of individuals phenotyped either for the vertical diameter of eyes to the width of the vertex between eyes (light gray) or for the nymphal viability (dark gray) (i.e., the minimum and maximum sample size for this dataset, respectively). We show median and interquartile range (*y*‐axis) of heritability estimates (a) and of *S*‐values for the model with an additive genetic variance (b) as a function of simulated heritability (*x*‐axis). The horizontal dotted line depicts the 4 bits of information against the null hypothesis (conveyed by a *p*‐value of .05)

“+” indicates an increase, “–” indicates a decrease, and “.” an absence of change of the phenotypic mean (*μ*) or of the evolvability measure (*I*_A_; i.e., mean‐standardized additive genetic variance) from the warm environment to the cold environment. When changes were significant (i.e., when models including a *V*_A_ effect were significant in a single environment, see Tables 2 and 3, or when there was no overlap between *V*_A_ compatibility intervals of the warm and cold environments, see Figure 3), we also reported in parentheses the natural logarithmic changes between optimal and stressful environments of *μ* and *I*_A_. 1. Sword et al. (2000); 2. Despland and Simpson (2005); 3. Simpson and Sword (2009); 4. Wilson et al. (2001); 5. Wilson et al. (2002); 6. Ott and Rogers (2010); 7. Roonwal (1947); 8. Esperk et al. (2007); 9. Honěk (1993).

^a^ To our knowledge, there is no direct evidence for a relationship between the variation in allometric wing length and flight performance in the desert locust. However, wing allometry (in particular length, loading and aspect ratio) have been related to flight performance in insects (Berwaerts et al., 2002 and references within).

^b^ See Information Box 2 for rationale for the presumed relationship of traits with fitness.

### 3.3 Statistical power of our quantitative genetic analysis

Our statistical power to detect additive genetic variation using our pedigree was low, except for high levels of heritability: The interquartile range of *S*‐value was fully above the 4 bits of information against the null hypothesis (conveyed by a *p*‐value of 0.05) for *h* ^2^ = 0.25 and 0.5 for the maximum (243) and minimum (108) sample sizes studied, respectively (Figure 3b). The power analysis also showed that heritability estimates were slight downwardly biased for both sample sizes (Figure 3a). In the presence of low levels of maternal effects, *h* ^2^ estimates tended to be inflated by a constant of 0.2 (Section S6 in the Supporting Information), in agreement with other published simulation studies (Kruuk & Hadfield, 2007; de Villemereuil et al., 2012). Overall, our statistical power remains low and our experimental procedure is conservative, even for *h* ^2^ estimates of evolutionary significance (e.g., 0.25). Note that, for any given sample size, our experimental design (large family size; more sires than dams by sires) is adequate for estimating low levels of heritability (Lynch & Walsh, 1998; Robertson, 1959).

## 4 DISCUSSION

In the context of global change, there is growing interest in understanding the potential of organisms to adapt to environments deviating from their optimal range. Sublethal stresses due to environmental variation within natural geographical or seasonal ranges may affect individuals' traits related to fitness (Dudley, 1961; Hamilton, 1936, 1950; Hoste et al., 2002), impact population dynamics, and shift future phenology and distribution of species with respect to current conditions. While it is clear that environmental stress can change additive genetic variation, the magnitude and direction of these changes are debated and may vary across traits within populations. In order to further address this fundamental issue, we investigated in the laboratory how the genetic parameters of 10 traits with different levels of proximity to fitness responded to a lifetime sublethal thermal stress in a population of the desert locust. In this ectotherm at the top end of the thermal tolerance spectrum (Maeno et al., 2021), our study explored as a thermal stress a low temperature that reflects winter local conditions. While our laboratory environments did not capture the full microclimatic variability from deserts and did not allow either for behavioral opportunities (e.g., warm‐up by basking in the sun; Maeno et al., 2021), they are likely to capture most of the cold stress related to seasonal variation. In any case, the imposed low temperature lengthened the development time up to three times (i.e., range of 20–59 days), costs of which are likely to reduce the fitness of individuals in nature (e.g., lower adult survival rate, mate availability, and reproductive success; Stearns, 1992).

We show that heritability remains mostly constant and no hidden additive genetic variance was revealed with environmental stress for the traits with strong and direct impact on fitness: nymphal viability, adult body weight (approximated by maximal nymphal body weight), and the dispersal‐related trait (approximated by allometric wing size). We did not observe either a stress‐induced increase in environmental variation (captured by *V*_R_), as is sometimes observed for similar traits in other species (Charmantier & Garant, 2005; Rowinski & Rogell, 2017). These traits also showed constant mean phenotypic values and negligible additive genetic variance across thermal environments. Thus, the absence of change between optimal and stressful environments for these three traits could also be partly explained by the fact that it is harder to detect for smaller values of genetic parameters (Charmantier & Garant, 2005). Nevertheless, our results are in accordance with these three traits being direct targets of strong stabilizing selection, as it is expected for traits tightly linked to individual fitness being genetically canalized, thereby becoming invariant in the face of environmental and genetic perturbations (Stearns & Kawecki, 1994). While this result is in agreement with most previous studies in insects for the body weight at adult emergence (see Pélissié et al., 2016), a high level of additive variance has been observed for dispersal‐related traits within Orthopteran populations (reviewed in Saastamoinen et al., 2013). However, these studies focused on temperate species that buffer seasonal variability through dormancy traits (e.g., diapause), lowering overall selection pressures on dispersal traits. In contrast, seasonal dispersal allows the desert locust to exploit newly available resources and survive in its erratic arid and semiarid habitat. Quantitative genetic estimates support that dispersal‐related traits might be under stabilizing selection in the desert locust, favoring a single migratory phenotype regardless of environmental change.

Conversely, we show that stressful conditions exposed a quantifiable amount of additive genetic variance for the three morphological traits more distantly linked with fitness in our laboratory environments: the nymphal green pigmentation, the ratio of the femur length over the head width, and the ratio related to the eye in the immature adult. For these traits, the change seems to be large enough to be detectable, even if the genetic parameters were rather small in the benign environment. These morphological traits serve ecological functions useful in other dimensions than the thermal environment: Head and eye allometries are known to be implicated in interindividual interactions under high population densities (Ott & Rogers, 2010; Roonwal, 1947), while green pigmentation is involved in protection of solitarious individuals from predators by homochromy (Sword et al., 2000; Despland & Simpson, 2005 and Simpson & Sword, 2009). Accordingly, these morphological traits did not show phenotypic plasticity in response to temperature, unlike in response to population density (see Table S1 in Section S1 in the Supporting Information).

Finally, four traits presumed to have evolved to compensate for the negative effects of a small hatchling size or adverse growth conditions (growth rate, development time, extramolting, and melanin pigmentation) showed both phenotypic plasticity in response to temperature and significant *V*_A_, in agreement with previous studies (Nolte, 1962, 1965, 1966; Pélissié et al., 2016). Thermal stress did not affect the expression of *V*_A_ in a consistent manner, as our results indicate either an absence of change or a change of moderate magnitude. Indeed, growth rate showed a decrease in *V*_A_ and *I*_A_ in the cold treatment, while development time was the single of the compensatory traits to unmask some amount of additive genetic variation with stress. This observation of mixed responses, never on a large scale, rather corroborates research from Wood and Brodie (2015) and can be predicted under evolutionary theory of phenotypic plasticity. First, although an increase in *V*_A_ for plastic traits can be triggered by fitness relocation under environmental stress, it strongly depends on the degree to which initial plasticity was adaptive (Ghalambor et al., 2015). In our study, both the probability to undergo an extramolt and the development time are predicted to exhibit adaptive plasticity, as environmentally induced phenotypes are altered in a direction that favors increased fitness (Information Box S2 in the Supporting Information). In contrast, maladaptive plasticity may explain the lower values for both mean and additive genetic variance of the intra‐instar growth rate under our cold treatment (Information Box S2 in the Supporting Information).

Second, the release of genetic additive variance for adaptive plastic traits depends on the intensity at which phenotypic compensation is compromised in the stressful environment (Paaby & Rockman, 2014; Schlichting, 2008). In our study, while the compensation for the lowered intra‐instar growth was enough for individuals to reach a similar adult body weight on average, we nevertheless observed marginal maladaptive responses under the cold treatment: A few nymphs never molted to the adult stage trapped in a juvenile stage until death (results not shown). These maladaptive phenotypes in development time under stress were expectedly accompanied by a release of hidden additive genetic variation (Paaby & Rockman, 2014; Schlichting, 2008). Similarly, a few nymphs showed a high production of melanin under cold stress, with a negative impact in all life history components, including adult body weight (i.e., negative significant phenotypic correlations). Whether melanic plasticity was adaptive in ancestral environments is unknown (Information Box S2 in the Supporting Information). However, the negative relationship between the direction of plasticity and the phenotypic optimum at least suggests that it does seem so under cold stress (Prokkola et al., 2013; Roff & Fairbairn, 2013).

Third, constant *V*_A_ estimates for plastic traits other than development time can be explained by the genetic variance–covariance matrix in which these traits evolved (Ellers & Liefting, 2015; Paaby & Rockman, 2014; Schlichting, 2008). Notably, plastic traits may depend on the same developmental pathway (Snell‐Rood et al., 2010), which is likely to be the case for growth rate, extramolt strategy, and development time, which all act as modules of adjustment of adult body size. This idea is in accordance with significant phenotypic correlations between these three growth traits in our data (Section S5 in the Supporting Information). Unfortunately, we could not investigate the importance of genetic correlations among traits, due to a lack of statistical power. In any case, our study drew attention to the importance of considering separately canalized and plastic traits when studying the effects of environmental stress on fitness‐related traits.

Altogether, our results support the hypothesis that the level of connection of a trait to individual fitness determines its potential level of cryptic genetic variation in optimal environmental conditions, which can be uncovered by exposing individuals to environmental stress during development. This conclusion is coherent with most stress evolution meta‐analyses: We confirmed previous evidence for a substantial increase in additive genetic variation with environmental stress for the traits least expected to contribute to fitness in studied environments (Hoffmann and Merilä's (1999) meta‐analysis; De Moed et al., 1997; Charmantier and Garant's (2005) meta‐analysis; Saxon et al., 2018 and Fischer et al., 2021). Our findings suggest that the lack of agreement with other studies (e.g., Rowinski & Rogell, 2017) and the lack of consensus in literature can at least partly be explained by a trait categorization (life history vs. morphology) across populations and species that is way too loose and may fail to capture correlation with fitness. For example, and in agreement with known traits' evolutionary significance in the desert locust, our results indicated that a body shape trait related to flight performance was closely linked to fitness through canalization across the natural range of environments, and a body pigmentation trait related to melanin production was indirectly linked to fitness through developmental plasticity. Both fitness‐related morphological traits showed an expression of additive genetic variation insensitive to the environmental stress experienced by the population.

Our conclusions should, however, be evaluated with caution, due to our limited sample size (i.e., 8 paternal half‐sibling families, 15 maternal full‐sibling families, and 108–243 measured individuals within thermal treatments). In particular, we might have been able to obtain statistical support for a *V*_A_ effect only in cases where *V*_A_ was large (see “effect size inflation” in Trafimow et al., 2018). A higher sample (e.g., of over 1,000 individuals) would have increased our statistical power to reveal a *V*_A_ effect within thermal environments, increased the accuracy of our *h* ^2^ estimates, and allowed for the estimations of genetic correlations across temperatures. Unfortunately, although the methods of rearing locusts in isolation have been optimized over the years, the intensity of labor involved (i.e., due to large body size, long life cycle, uneasy handling, and low survival and reproductive parameters) strongly limits the number of individuals that can be reared, monitored, and measured under a certain family structure (Hoste et al., 2002; Pélissié et al., 2016; Roessingh et al., 1993).

Because these logistical constraints are common, most animal stress evolution studies have focused on a few model organisms (e.g., *Drosophila* sp.; Wood & Brodie, 2015) for their ease of sampling, rearing, and phenotyping in large numbers. However, trait evolutionary responses, especially to global change, should not be restricted to a few taxonomic groups: Some questions and hypotheses can be addressed only with nonmodel organisms, and the generality of conclusions can be ascertained only by moving away from model organisms. In this context, we argue that while the desert locust does not offer the advantages of tried‐and‐true model organisms, its rearing and individual phenotyping are feasible, allowing to study its unique ecology and evolution. Furthermore, global change research urges caution from single‐trait analyses because species' traits often displayed contrasted responses to temperature variation (e.g., Urban et al., 2016). Yet, most evolutionary studies on model organisms support trends based on few, easy‐to‐phenotype traits (Rowinski & Rogell, 2017; Wood & Brodie, 2015). Our in‐depth study of multiple and contrasted known traits in the desert locust allows to draw nuanced conclusions about trait responses to thermal stress, which partially offsets our sample size constraints.

By bringing insights in the potential evolutionary trajectory of traits involved in demographic processes (i.e., growth, survival, and dispersal) under environmental stress, our results should help predict more accurately how global change might affect populations' dynamics and invasions (Maeno et al., 2021; Meynard et al., 2017, 2020). At a short timescale, the heritable variation that is released under the more intense selection regime of stressful conditions provides a reservoir of standing genetic variation that can contribute to the adaptation of populations (Endler, 1986). Whether these environmentally induced changes in additive genetic variance can have longer‐term evolutionary effects in populations certainly depends on the fraction of traits that exhibit this behavior, on the structure of the genetic variance–covariance matrix (especially, genetic correlations), and on the heterogeneity of selection among environments experienced in nature. Our results indicate that adaptive potential may be boosted under environmental stress, both for some highly responsive traits loosely linked to fitness and for a few mildly responsive adaptive plastic traits in which phenotypic compensation is compromised (e.g., development time in our study). It is noteworthy that traits exhibiting adaptive plasticity are predicted to release new additive genetic variation (Ellers & Liefting, 2015; Paaby & Rockman, 2014; Schlichting, 2008), while such plasticity reduces adaptive evolution under new selective environments, as if it could compensate (Ghalambor et al., 2015). In contrast, the early stages of selection in new environments are predicted to potentiate rapid adaptive evolution of maladaptive plasticity (e.g., growth rate in our study) (Ghalambor et al., 2015). It is reasonable to hypothesize that this general trend could hold for other invasive or pest species that often experience highly variable environments (both spatially and/or temporally) and exhibit phenotypic plasticity for a wide range of traits (e.g., Horkova & Kovac, 2014; Niu et al., 2013). As always, multiple lines of evidence, from further experimental work and on a wider range of taxa, are needed to generalize this pattern and potentially uncover universal drivers of trait evolution (Tong, 2019).

Since our study is the first to estimate the genetic contribution to the phenotypic expression of key phase traits in a locust, our results should also beneficiate models of phase polyphenism evolution (Ayali, 2019). Although additive genetic variation was not visible for most morphological traits involved in phase polyphenism under standard conditions, ecologically relevant cold stress released cryptic genetic variation for most of them, including the most discriminative of all morphometric phase ratios (i.e., ratio of the length of the hind femur to the maximum width of the head). Traits related to phenology have already been detected to respond to ongoing climate change in insect populations (Lehmann et al., 2020). Accordingly, development time was the single life history trait to unmask genetic variation in the desert locust, exposing it to stronger selection under environmental thermal stress, a potentially crucial driver of phase polyphenism evolution in nature. Genetic correlations between phase traits related to life history and morphology may, however, constrain future adaptive evolution. In this respect, we note that slow‐growing (and heavier) nymphs had values toward those of the solitarious morph for all morphological phase traits (i.e., shorter elytra, smaller heads, smaller eyes, and more green pigmentation) but for the amount of melanin pigmentation.

A next prospect would be to explore whether the main driver of ongoing global change, increasing temperatures, would also expose same traits to selection. Air temperature already exceeds the desert locust optimal body temperature of 40℃, at least in the hot hyperarid regions of its northern range (i.e., Sahara, Sahel, Thar, and Arabian deserts) and during hours around solar noon (Maeno et al., 2021). In face of such a heat stress, the species had evolved an upper thermal limit for survival of 51℃ and multiple thermoregulatory behaviors to cool down (e.g., stilting on the hot ground, moving to the shade, and perching on shrubs; Maeno et al., 2021). Since ongoing global warming may increase the frequency, intensity, and/or duration of daily heat waves (IPCC, 2013), next evaluations of its influences in the desert locust should be conducted under opportunities for microenvironments and under fluctuating temperatures, which explore large daily temperature change (Lehmann et al., 2020).

## CONFLICT OF INTEREST

The authors declare no conflict of interest.

## AUTHOR CONTRIBUTIONS

**Marie‐Pierre Chapuis:** Conceptualization (equal); funding acquisition (equal); investigation (supporting); methodology (equal); supervision (equal); writing‐original draft (lead). **Benjamin Pélissié:** Conceptualization (equal); data curation (lead); investigation (lead); methodology (equal); writing‐review & editing (equal). **Cyril Piou:** Conceptualization (equal); data curation (supporting); funding acquisition (equal); investigation (supporting); methodology (equal); supervision (equal). **Floriane Chardonnet:** Data curation (equal); methodology (supporting). **Christine Pagès:** Investigation (equal). **Antoine Foucart:** Investigation (supporting). **Elodie Chapuis:** Methodology (supporting). **Hélène Jourdan‐Pineau:** Data curation (supporting); methodology (equal); writing‐review & editing (equal).

## Supporting information

Supplementary Material

## Data Availability

Phenotypic and pedigree data and R scripts for all analyses are publicly available on the CIRAD Dataverse repository (https://dataverse.cirad.fr/) at https://doi.org/10.18167/DVN1/S735JB.
