# Supplementary material for: Additive genetic variance for traits least related to fitness increases with environmental stress in the desert locust, Schistocerca gregaria
Source: Ecol Evol. 2021 Sep 21;11(20):13930–47. doi: 10.1002/ece3.8099 (PMC8525110; doi:10.1002/ece3.8099)
Supplement: Supplementary file 1 — Supplementary Material [file ECE3-11-13930-s001.pdf]

Supporting information for:

"Additive genetic variance for traits least related to fitness increases with environmental stress in the desert locust, *Schistocerca gregaria*"

Marie-Pierre Chapuis[1,2], Benjamin Pélissié[1,2,3*], Cyril Piou[1,2*], Floriane Chardonnet[1,2], Christine Pagès[4], Antoine Foucart[1,2], Elodie Chapuis[5,6], Hélène Jourdan-Pineau[1,2,6,7,8]

[1]CIRAD, CBGP, Montpellier, France
[2]CBGP, CIRAD, Montpellier SupAgro, INRA, IRD, Univ Montpellier, Montpellier, France
[3]University of Nebraska at Kearney, Department of Biology, Kearney NE, 98849, USA
[4]CIRAD, UPR B-AMR, F-34398, Montpellier, France
[5]MIVEGEC, Université de Montpellier, CNRS, IRD, Montpellier, France
[6] CIRAD, UMR PVBMT, F-97410 Saint-Pierre, La Réunion, France
[7]CIRAD, UMR ASTRE, F-34398 Montpellier, France
[8]ASTRE, Univ Montpellier, CIRAD, INRA, Montpellier, France
[*] equal contribution

Contents:

1- **Procedures for minimizing parental environmental effects in the experiment**
2- **Pedigree visualisation**
3- **Methods for color and shape data**
4- **Fixed effects of temperature, sex, extra-molting, and hatching weight on the 10 traits measured in this study**
5- **Pairwise phenotypic correlations**
6- **Simulation analysis of the sensitivity of our quantitative genetics analysis to the presence of a low maternal effect**

Literature cited

1- Procedures for minimizing parental environmental effects in the experiment

Although our dataset was obtained from a nature-derived laboratory population, our protocol was designed to minimize non-genetic parental effects on our genetic estimates. First, we removed the main environmental source of parental effects, *i.e.* crowding, for four successive generations, by rearing individuals under the same isolation conditions in 1L individual plastic cages. Indeed, the desert locust can experience phase polyphenism: a suite of profound, transgenerational, and plastic changes, in response to dramatic increases in local population densities, caused by scattered heavy rains that result in local concentration of food resources (Pener & Simpson, 2009). Table S1 summarizes both literature-based evidence for parental and lifetime effects of population density on the 10 studied traits. The main hypothesis explaining the proximal causes of maternal effects related to population density, involves a crowd-mediated maternal factor either controlling primary egg size (and thus the amount of yolk) which in turn influences hatchling size and color (Maeno & Tanaka, 2010), or released from the reproductive accessory glands in the egg foam and influencing offspring black pigmentation and behavior (McCaffery, Simpson, Islam, & Roessingh, 1998; Simpson & Miller, 2007).

Second, we standardized rearing and maintenance during the whole experiment, in order to equalize parental and fore-parental environments across individuals, within our population. Standardized rearing conditions were a temperature at 34.0°C, 55% humidity, photoperiod of 12/12 hours, and ad libitum feeding with fresh wheat shoots and bran for three successive generations prior to phenotypic measurements. Desert locust populations experience highly variable thermal conditions in the wild (Roffey & Magor, 2003), with air temperature in deserts varying drastically between seasons (from an average of about 30°C to

15°C) and between day and night (from over 50°C to below freezing; Ward 2009). Table S1 summarizes literature-based evidence for parental and lifetime phenotypic effects of temperature, humidity and food on the 10 studied traits. Overall, parental effects induced by these environmental factors are scarce in comparison to those mediated by population density (or gregariousness).

Through this strict standardization of the rearing conditions during four laboratory generations, the remaining parental effects should be strongly restricted to pure genetic variation among parents, (hard to control) micro-environmental variation and to gene-by-environment interactions. Furthermore, phenotypic measurements were performed on integrative growth traits and early adult traits that are less sensitive to maternal effects than traits involved in early development (and survival). Indeed, maternal effects are expected to be larger for early offspring traits than for late traits, even if they can persist into adulthood (McAdam et al., 2014). Although whether maternal effects detected in hatchlings would persist in later stages is unknown, it has been shown in locusts that the colour of the hatchlings can change drastically in the second instar, depending on the rearing density experienced during the first instar (Injeyan & Tobe, 1981; Tanaka & Maeno, 2006). This suggests that the susceptibility to maternal effects of early and late nymphal development is significantly decoupled in *S. gregaria*.

Finally, we used a half-sib / full-sib quantitative genetics design with a paternal crossing scheme, which allows for estimates of V_A that are not inflated by common environmental effects, especially maternal effects (*e.g.*, nutritional resources provided in the egg by the mother). With all these precautions taken, we can safely assume that our experimental design was efficient in minimizing non-genetic parental effects in our dataset. In this context, adding a maternal effect (cancelled out by an experimental control) in the animal model would lead to statistical overfitting: a model too complex for the data captures too

much random noise, is expected to perform poorly, and can lead to unreliable estimates. In other words, since the animal model specifying only a genetic effect captures most of the complexity of our data, it will make the best prediction. Under this modelling strategy, we are only limited by the size of our dataset, which may generate some measurement errors (see the simulation-based power analysis, especially Figure 3, in the main text).

Table S1. Literature-based evidence for lifetime and parental effects of stressful environments (*i.e.*, low temperature, gregarious density, low humidity and low quality of food) on traits measured in this study

Category of traits	Mediated by	Lifetime effect (phenotypic plasticity)	Parental effect (on early stage)
Morphological phase traits			
	Temperature	more melanin marks[1,2] , shorter wings and larger heads [3,4]	more melanin marks[1]
	Density	more melanin marks[5-7], lower green background[4], longer wings, larger heads, and smaller eyes [4,8]	more melanin marks[2,5,9-14], larger heads [9,13]
	Humidity	longer wings and larger heads [3,4]	NA
	Food	longer wings and larger heads [15,16]	less melanin marks[4]
Nymphal life history traits			
	Temperature	smaller body size[8], slower growth[17-21], lower viability[17-19,22]	NA
	Density	smaller body size[5,6,,23], slower growth[5], less extra-molting[24]	larger body size[12,23], slower growth[5], less extra-molting[13]
	Humidity	slower growth[17,18], lower viability[17,18]	NA

	Food	smaller body size[15,16,25], slower growth[16,25], more extra-molting[16]	smaller body size[16]

The directional changes shown here are from optimal to stressful environments (*i.e.* gregarious density, low temperature, low humidity and low quality of food). Note that there are interaction terms between temperature and humidity not detailed here. Temperature, density, humidity and food were controlled for in our quantitative genetics experiment. Parental influences summarized here concern early stages while in our study, all morphological phase traits were measured in late stages. Body shape is assessed through the four morphometric ratios *E/F*, *F/C*, *F/V* and *O/V* with *E*: Length of the fore wing; *F*: Length of the hind femur; *C*: Maximum width of the head; *H*: Height of the pronotum; *P*: Length of the pronotum; *O*: Vertical diameter of eyes; *V*: Width of the vertex between eyes (see main text and section 2 of the Supporting Information for further details). NA: no data, controversial data or no effect. 1. Elliot *et al.*, 2003; 2. Nolte, 1962; 3. Dudley, 1964; 4. Stower, Davies & Jones, 1960 ; 5. Hunter-Jones, 1958; 6. Nickerson, 1956; 7. Nolte, 1965; 8. Dirsh, 1953; 9. Bouaichi & Simpson, 2003; 10. Islam *et al.*, 1994a; 11. Islam *et al.*, 1994b 12. Maeno & Tanaka, 2010; 13. Maeno & Tanaka, 2009; 14. McCaffery *et al.*, 1998;; 15. Maeno & Tanaka, 2011; 16. Manchanda, Sachan & Rathore, 1980; 17. Hamilton, 1936; 18. Hamilton & others, 1950; 19. Husain & Ahmad, 1936; 20. Gündüz & Gülel, 2002; 21. Wardhaugh *et al.*, 1969; 22. Papillon, 1968a; 23. Papillon, 1968b; 24. Maeno & Tanaka, 2008; 25. Van Huis *et al.*, 2008.

2-Pedigree visualization

Figure S1 shows our pedigree for the 483 G5 individuals. Five laboratory generations are depicted, including the first generation (G1) issued from the nine egg-pods laid by wild females collected in Mauritania in December 2010. The parentage relationships of these G1 offspring were inferred based on multi-locus genotyping as described in Pélissié et al. (2016). Other laboratory generations are the three generations of environmental standardization (G2 to G4) and the 5th generation of thermal treatments and phenotypic measurements. While the number of offspring varied substantially between families and between juvenile (2 to 29 offspring/family/trait/treatment) and adult (1 to 17 offspring/family/trait/treatment) traits, the high nymphal mortality (46%) was random across our sample and the family structure remained constant between traits and treatments (*i.e.*, 13-15 dams and 8 sires).

Figure S1. Drawing of the pedigree for the 483 G5 individuals.

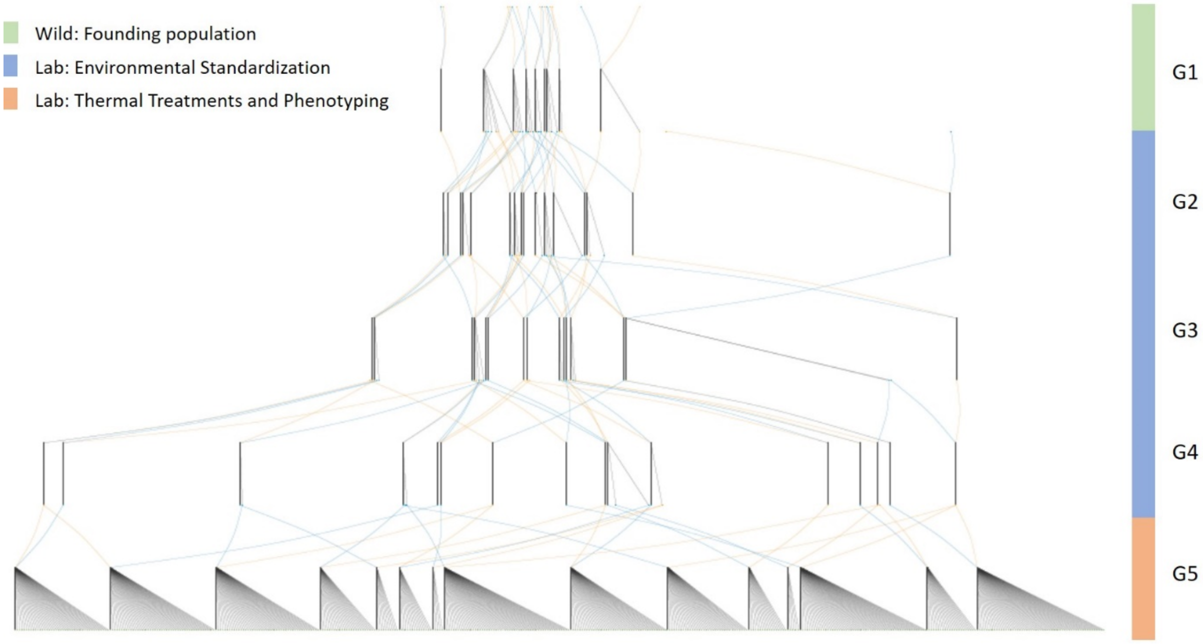

3- Methods for color and shape data

3.1- Methods for nymphal pronotum color data

Last-instar nymphs were anesthetized with CO_2 and photographed in lateral view. Pictures were corrected for white balance, using the PhotoFiltre 7© software improved by an external module (plugin “wbadjust”), and analyzed using ImageJ v1.52a (Abràmoff *et al.*, 2004). We selected the whole lateral surface of the pronotum, using the “Polygone selection” function. The pronotal color pattern is known to correlate to the color pattern of the rest of the body (*e.g.*, head, thorax) (Hunter-Jones 1958). We measured each color channel (RGB) as a 8–bit display mean value in the range 0–255, using the “Color Histogram” function, and calculated the percentage of green color (*Green Pigmentation*) as G/(R+G+B) (see Fig.S2 for illustrations). We differentiated the set of pixels for which brightness was in the 25% lower range (*i.e.*, 0–64) from the background, using the “Color Threshold” function, and calculated the percentage of dark color (*Dark Pigmentation*) as number of dark pixels/ number of total pixels (see Fig.S2 for illustrations). In order to provide a better approximation to the Gaussian distribution for statistical analysis, we used a logit transformation on the dark pixel data: log((number of dark pixels)/(number of total pixels - number of dark pixels)). In order to avoid zeros, we preliminary added half a pixel to numbers of dark pixels and of total pixels.

Figure S2. Illustrations of melanin and background coloration of nymphs.

	Beige color background	Green color background
**No marks**	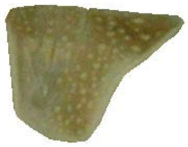<br>*Green Pigmentation* = 0.36<br>*Dark Pigmentation* = 0.00	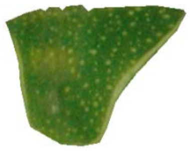<br>*Green Pigmentation* = 0.49<br>*Dark Pigmentation* = 0.00
**Melanin marks**	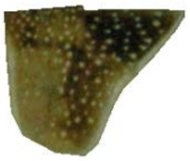<br>*Green Pigmentation* = 0.37<br>*Dark Pigmentation* = 0.27	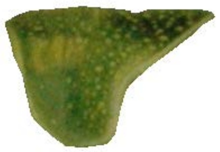<br>*Green Pigmentation* = 0.43<br>*Dark Pigmentation* = 0.06

3.2- Methods for adult body shape data

Adults were anesthetized with CO_2 and photographed in both lateral and dorsal views (Fig. S3) along with a scale of 30mm. Pictures were analyzed with the software ImageJ© v1.52a (Abràmoff et al. 2004). We measured five morphometric distances in adult locusts as shown in Fig. S4: the elytron length (*E*), the length of the hind femur (*F*), and the vertical diameter of eyes (*O*), the maximum width of the head (*C*), and the width of the vertex between eyes (*V*).

Figure S3. Illustrations of an adult in lateral and dorsal views.

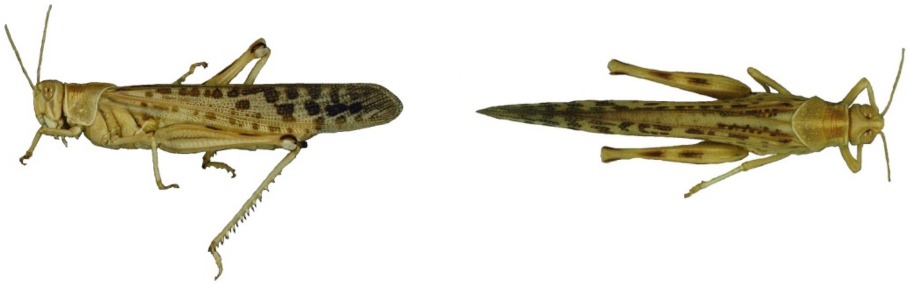

Figure S4. Illustrations of measurements used for calculating shape variables (*i.e.*, morphometric ratios; from Dirsh, 1953).

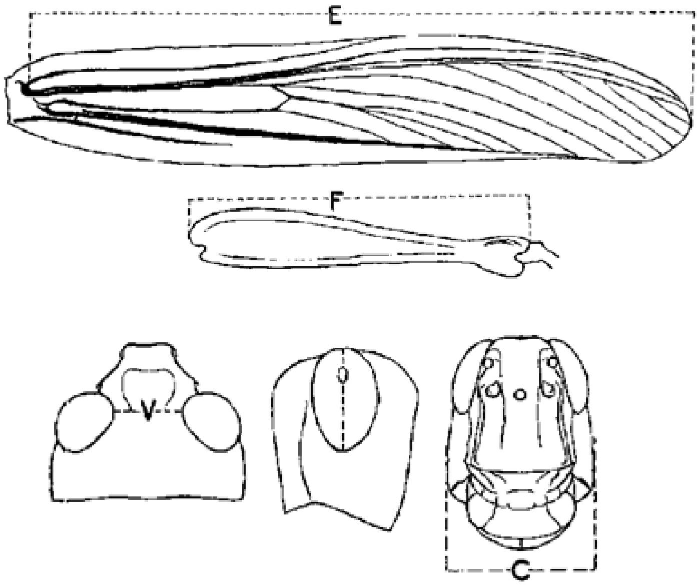

4- Fixed effects of temperature, sex, extra-molting, and hatching weight on the 10 traits measured in this study

Mean ±SD for each level and of categorical factors and statistical values for each factor are presented in Table S2 and S3, respectively. Effects of temperature on color, shape, growth and nymphal life history traits are described in the main document. We described below other effects than temperature associated 4 bits of information against the null hypothesis (Greenland, 2019). Males had a lower body size, green pigmentation and probability of extra-molting, as well as larger eyes and a higher dark pigmentation than females. *Extra-Molting* and the interaction between *Sex* and *Extra-Molting* also had an effect on maximal nymphal weight: female and extra-molting individuals were bigger, and the latter effect was much stronger for females. Individuals with a normal growth had a higher amount of dark pigmentation than individuals that performed an extra-molt. *Hatching Weight* had a negative effect on the probability of extra molting and on the growth rate during the first and the third nymphal instar only, meaning that smaller offspring, independently of their sex or molt strategy, displayed a higher growth rate. *Hatching Weight* had also a small effect on the allometric wing length (negative effect) and on the allometric head size of the individuals that underwent an extra-molt only (positive effect). The observed *P*-values also suggested that data was congruent with an absence of effect of *Hatching Weight* on the maximal nymphal weight. The results on life history traits mostly agree with results from Pélissié *et al.* (2016).

Table S2. Description of the experimental dataset: sample size (N), mean and standard deviation (SD) for the 10 measured traits as a function of temperature, sex, and extra-molting (if applicable).

A) Morphological phase traits

		Cold environment				Warm environment			
		Female		Male		Female		Male	
	Extra-Molting	0	1	0	1	0	1	0	1
Green Pigmentation	N	11	46	34	23	8	24	21	21
	**Mean**	**0.441**	**0.454**	**0.434**	**0.439**	**0.458**	**0.456**	**0.412**	**0.446**
	SD	0.031	0.033	0.041	0.03	0.02	0.034	0.037	0.035
Dark Pigmentation	**N**	11	46	36	24	8	25	21	21
	**Mean**	**0.0475**	**0.0131**	**0.0304**	**0.0174**	**0.0004**	**0.0023**	**0.0024**	**0.0082**
	SD	0.1497	0.0374	0.0683	0.0233	0.0007	0.0061	0.0044	0.0142
	**N**	11	39	35	24	14	25	47	18
E/F	**Mean**	**1.993**	**2.029**	**2.026**	**2.012**	**2.102**	**2.087**	**2.049**	**2.026**
	SD	0.066	0.057	0.063	0.096	0.078	0.072	0.069	0.058
F/C	N	11	42	35	23	14	27	47	19
	**Mean**	**3.99**	**3.931**	**3.759**	**3.789**	**3.896**	**3.908**	**3.859**	**3.907**
	SD	0.187	0.218	0.171	0.247	0.23	0.175	0.22	0.164
O/V	N	11	39	35	23	14	25	47	18
	**Mean**	**1.958**	**1.946**	**2.094**	**2.012**	**2.002**	**2.083**	**2.261**	**2.323**
	SD	0.121	0.307	0.293	0.28	0.229	0.23	0.259	0.226

E: Length of the fore wing; *F*: Length of the hind femur; *C*: Maximum width of the head; *H*: Height of the pronotum; *P*: Length of the pronotum; *O*: Vertical diameter of eyes; *V*: Width of the vertex between eyes.

B) Life history traits (nymphal growth and viability)

		Cold environment				Warm environment			
		Female		Male		Female		Male	
	Extra-Molting	0	1	0	1	0	1	0	1
Growth Rate	N	13	53	37	27	19	33	54	28
	**Mean**	**0.155**	**0.162**	**0.161**	**0.165**	**0.282**	**0.303**	**0.295**	**0.287**
	SD	0.024	0.017	0.018	0.021	0.043	0.047	0.047	0.042
Extra-Molting	N	66		64		52		82	
	**Freq**	**0.803**		**0.422**		**0.635**		**0.341**	
Development Time (days)	N	12	47	37	25	15	30	50	23
	**Mean**	**44.333**	**50.83**	**43.568**	**48.920**	**23.400**	**25.067**	**22.340**	**24.870**
	SD	2.270	2.815	2.230	2.532	2.197	0.907	1.303	1.961
Maximal Nymphal Weight (grams)	N	12	50	37	25	17	32	52	27
	**Mean**	**1.870**	**2.274**	**1.307**	**1.532**	**1.913**	**2.291**	**1.271**	**1.414**
	SD	0.141	0.218	0.100	0.122	0.230	0.237	0.122	0.221
Nymphal Viability	N	243				240			
	**Freq**	**0.498**				**0.496**			

Table S3. Factors influencing morphological phase and nymphal life history traits in the desert locust.

Trait	Selected variables	F / χ^2*	*P*-value	*S*-value
**Morphological phase**				
Green Pigmentation	Intercept	119.44	<0.001	>9.966
	Temperature	0.53	0.467	1.099
	Sex	4.57	0.034	4.878
	Extra-molting	2.38	0.125	3.000
	Hatching weight	0.42	0.517	0.952
Dark Pigmentation	Intercept	86.56	<0.001	>9.966
	Temperature	27.82	<0.001	>9.966
	Sex	11.10	<0.001	>9.966
	Extra-molting	3.04	0.083	3.591
	Hatching weight	1.74	0.189	2.404
E/F	Intercept	850.65	<0.001	>9.966
	Temperature	0.22	0.637	0.651
	Sex	0.41	0.523	0.935
	Extra-molting	8.21	0.005	7.644
	Hatching weight	4.74	0.031	5.012
	Extra-molting: Hatching weight	10.22	0.002	8.966
F/C	Intercept	3494.17	<0.001	>9.966
	Temperature	3.06	0.082	3.608
	Sex	0.44	0.509	0.974
	Hatching weight	6.00	0.015	6.059
O/V	Intercept	1312.14	<0.001	>9.966
	Temperature	0.05	0.824	0.279
	Sex	19.06	<0.001	>9.966
	Extra-molting	1.54	0.217	2.204
**Life history**				
Growth Rate	Intercept	2056.22	<0.001	>9.966
	Temperature	923.41	<0.001	>9.966
	Hatching weight	23.07	<0.001	>9.966
Extra-Molting	Temperature	6.96	0.008	6.966
	Sex	11.61	<0.001	>9.966
	Hatching weight	12.70	<0.001	>9.966
Development Time	Intercept	195.52	<0.001	>9.966
	Temperature	36.23	<0.001	>9.966
	Sex	2.80	0.095	3.396

	Extra-molting	0.02	0.892	0.165
	Hatching weight	2.29	0.132	2.921
Maximal Nymphal Weight	Intercept	2742.00	<0.001	>9.966
	Temperature	0.47	0.494	1.017
	Sex	226.20	<0.001	>9.966
	Extra-molting	98.07	<0.001	>9.966
	Sex: Extra-molting	17.32	<0.001	>9.966
Nymphal Viability	Hatching weight	2.17	0.140	2.837

For each trait, selected variables came from linear models displaying the lowest AIC score, among all possible models from a full model (*i.e.*, containing all variables as well as every simple interaction between pairs of variables for a total of 15 terms) to a null model. We reported observed *P*-values and their Shannon information transforms *S*-value= $-\log_2$(*P*-value), which measure the degree of incompatibility between the data and the null hypothesis of the model. For the sake of simplicity, we reported interaction terms only if their two components and themselves had a *P*-value ≤ 0.05. The effects of *Sex* and *Extra-Molting* could not be assessed on *Nymphal Viability* since nymphs that died early in their development have not yet get the opportunity to be sexed nor to extra-molt.* Note that a generalized linear model with binomial distribution was used for *Extra-Molting* and *Nymphal Viability* (see χ^2 values), whereas a linear model was used for all other variables (see F values). *E*: Length of the fore wing; *F*: Length of the hind femur; *C*: Maximum width of the head; *H*: Height of the pronotum; *P*: Length of the pronotum; *O*: Vertical diameter of eyes; *V*: Width of the vertex between eyes.

5- Pairwise phenotypic correlations

Phenotypic correlations among color, shape, growth and nymphal life history traits are shown in Table S4. As for morphological phase traits, greener individuals developed smaller heads and smaller heads correlated with shorter wings. These results are similar with the response predicted in phase polyphenism. As for growth and life history traits of nymphs, and as often reported in insects, development time co-varied positively with body size and negatively with growth rate. The extra-molt strategy that allowed for greater body size represented a developmental compromise with development time (increased). Finally, fast-growing individuals (see traits *Growth Rate* and *Development Time*) and lighter last-instar nymphs (see traits *Maximal Nymphal Weight* and *Extra-molting*) had values of phase traits towards those of the gregarious morph, with longer elytra, larger heads, larger eyes and/or less green pigmentation. This is agreement with correlations predicted in phase polyphenism (see references in Table S1). The single exception was *Dark Pigmentation* that was associated with both a low body weight and a slow growth. These correlations were moderate, with absolute Pearson coefficient values ranging from 0.14 to 0.55. The single high value (>0.8) was found for the overall growth rate and development time, traits that mathematically derived from each other.

Table S4. Pearson's r rank coefficients (below diagonal) and *P*-values (above diagonal) for pairwise phenotypic correlations between traits.

	Green pigmentation	*Dark pigmentation*	*E/F*	*F/C*	*O/V*	*Growth Rate*	*Extra-Molting*	*Development Time*	*Maximal Nymphal Weight*
Green Pigmentation		-0.063	5.38E-01	**1.32E-02**	5.67E-01	9.71E-01	**6.54E-04**	5.56E-01	**6.39E-06**
Dark Pigmentation	0.127		4.21E-01	8.13E-02	5.84E-01	**3.03E-05**	5.82E-01	**7.59E-07**	**1.04E-02**
E/F	-0.049	-0.134		**6.94E-07**	9.38E-02	**4.23E-04**	6.53E-01	**2.58E-04**	1.72E-01
F/C	**0.193**	-0.111	**-0.333**		2.40E-01	2.03E-01	9.89E-02	5.14E-01	**7.59E-04**
O/V	-0.045	-0.184	-0.115	0.081		**4.23E-05**	**3.03E-02**	**4.27E-07**	**5.64E-05**
Growth Rate	-0.003	**-0.302**	**0.239**	0.087	**0.277**		9.95E-02	**0.00E+00**	3.50E-01
Extra-Molting	**0.244**	-0.040	-0.031	0.112	**-0.149**	-0.107		**9.02E-07**	0.00E+00
Development Time	0.044	**0.355**	**-0.248**	-0.045	**-0.338**	**-0.872**	0.311		**5.49E-05**
Maximal Nymphal Weight	**0.320**	**-0.184**	0.094	**0.226**	**-0.272**	-0.061	**0.554**	**0.257**	

Values were computed with the function rcorr of the Hmisc package in R (http://biostat.mc.vanderbilt.edu/wiki/Main/Hmisc). Nymphal viability could not be correlated with the other traits since dead nymphs were not measured for any other traits. *E*: Length of the fore wing; *F*: Length of the hind femur; *C*: Maximum width of the head; *H*: Height of the pronotum; *P*: Length of the pronotum; *O*: Vertical diameter of eyes; V: the width of the vertex between eyes.

6- Simulation analysis of the sensitivity of our quantitative genetics analysis to the presence of a low level of maternal effects.

Figure S5: Sensitivity of our quantitative genetics analysis to a low level of maternal effects. We show median and interquartile range (y-axis) for heritability estimates (A) and *S*-values for the model with an additive genetic variance (B) as a function of simulated heritability (x-axis). We set 11 levels of heritability (*i.e.*, 0. 0, 0.1, 0.2, 0.3, 0.4, 0.5, 0.6, 0.7, 0.8, 0.9, 1.0) and, for each level, we simulated 1,000 phenotypic datasets based on our experimental design at the low temperature, *i.e.* with exactly the same pedigree as for the subset of individuals phenotyped either for the morphometric ratio *O/V* (light grey) or the *Nymphal Viability* (dark grey) (*i.e.*, the minimum and maximum sample size for this dataset, respectively). The level of maternal effect was set to 0.1.

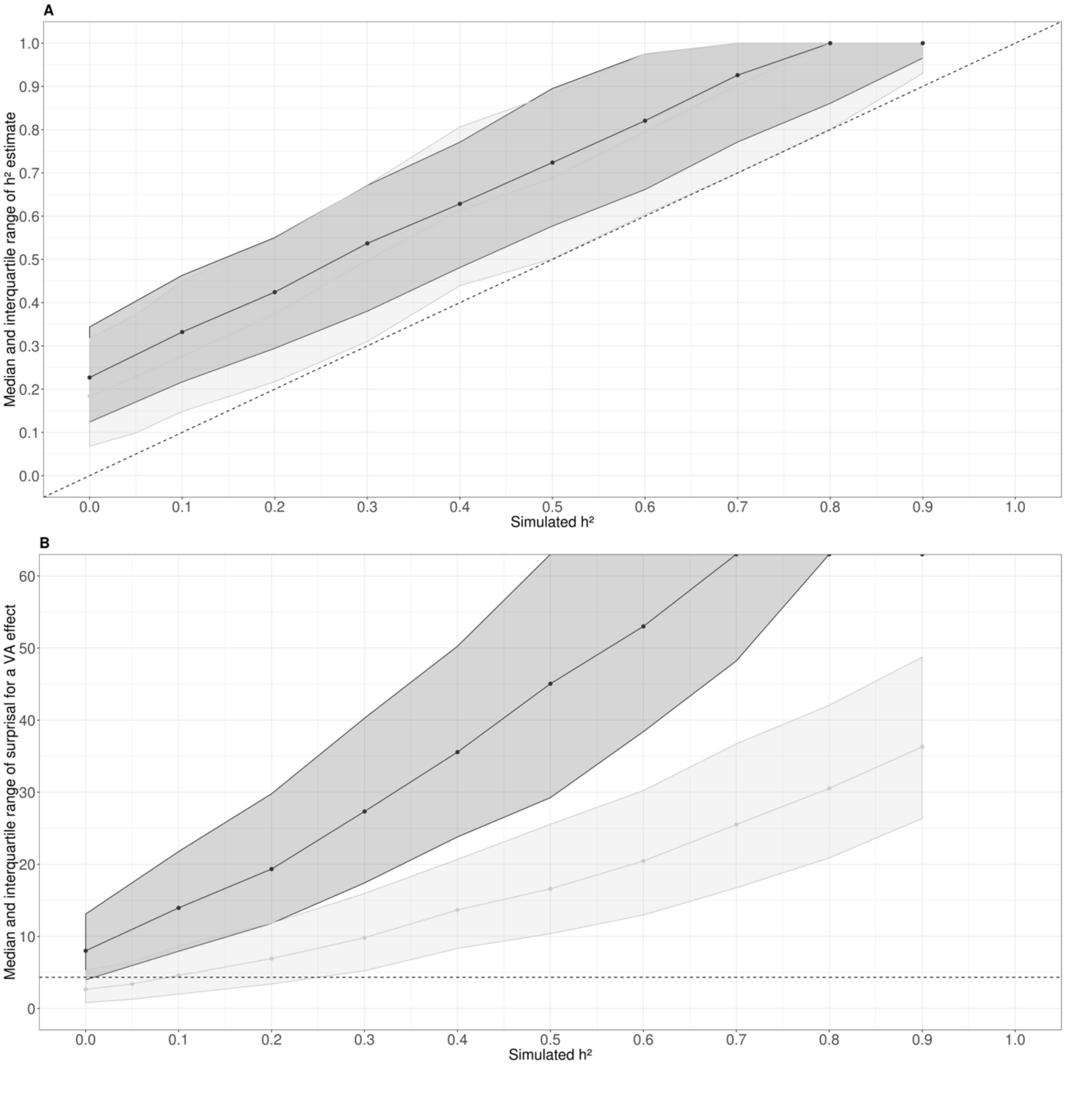

Literature cited

Abràmoff MD, Magalhães PJ, Ram SJ (2004) Image processing with image J. *Biophotonics International*, 11:36–41

Bouaichi A, Simpson SJ (2003) Density dependent accumulation of phase characteristics in a natural population of the desert locust *Schistocerca gregaria*. *Physiological Entomology*, 28:25–31

Dirsh VM (1953) Morphometrical studies on phases of the desert locust (*Schistocerca gregaria* Forskal). *Anti-Locust Bulletin*, 16:1–34

Dudley B (1964) The effects of temperature and humidity upon certain morphometric and colour characters of the desert locust (*Schistocerca gregaria* Forskal) reared under controlled conditions. *Transactions of the Royal Entomological Society of London*, 16:115–129

Elliot SL, Blanford S, Horton CM, Thomas MB (2003) Fever and phenotype: transgenerational effect of disease on desert locust phase state. *Ecology Letters*, 6:830–836

Greenland S (2019) Valid *P*-values behave exactly as they should: some misleading criticisms of P-values and their resolution with *S*-values. *The American Statistician*, 73, 106-114

Gündüz NEA, Gülel A (2002) Effect of temperature on development, sexual maturation time, food consumption and body weight of *Schistocerca gregaria* Forsk. (Orthoptera: Acrididae). Turkish *Journal of Zoology*, 26:223–227

Hadfield JD (2010) MCMC methods for multi-respoinse generalized linear mixed models: The MCMCglmm R package. *Journal of Statistical Software*, 33:1–22

Hamilton AG (1936) The relation of humidity and temperature to the development of three species of African locusts: *Locusta migratoria migratorioides* (R. & F.), *Schistocerca gregaria* (Forsk.), *Nomadacris septemfasciata* (Serv.). *Transactions of the Royal Entomological Society of London*, 85:1–60

Hamilton AG (1950) Further studies on the relation of humidity and temperature to the development of two species of African locusts. *Locusta migratoria migratorioides* (R. & F.) and *Schistocerca gregaria* (Forsk.). *Transactions of the Royal Entomological Society of London*, 101:1–58

Hunter-Jones P (1958) Laboratory studies on the inheritance of phase characters in locusts. *Anti-Locust Bulletin*, 29:1–32

Husain MA, Ahmad T (1936) Studies on *Schistocerca gregaria* Forsk, II: The biology of the desert locust with special reference to temperature. *Indian Journal of Agricultural Science*, 6

Injeyan HS, Tobe SS (1981). Phase polymorphism in *Schistocerca gregaria*: Reproductive parameters. *Journal of Insect Physiology*, 27, 97–102

Islam MS, Roessingh P, Simpson SJ, et al (1994a) Effects of population density experienced by parents during mating and oviposition on the phase of hatchling desert locusts, *Schistocerca gregaria*. *Proceedings of the Royal Society of London. Series B: Biological Sciences*, 257:93–98

Islam MS, Roessingh P, Simpson SJ, Mccaffery AR (1994b) Parental effects on the behavior and coloration of nymphs of the desert locust *Schistocerca gregaria*. *Journal of Insect Physiology*, 40:173–181

Maeno K, Tanaka S (2008) Phase-specific developmental and reproductive strategies in the desert locust. *Bulletin of Entomological Research*, 98:527–534

Maeno K, Tanaka S (2009) The trans-generational phase accumulation in the desert locust: Morphometric changes and extra molting. *Journal of Insect Physiology*, 55:1013–1020

Maeno K, Tanaka S (2010) Epigenetic transmission of phase in the desert locust, *Schistocerca gregaria*: determining the stage sensitive to crowding for the maternal determination of progeny characteristics. *Journal of Insect Physiology*, 56:1883–8

Maeno K, Tanaka S (2011) Phase-specific responses to different qualities of food in the desert locust, *Schistocerca gregaria*: Developmental , morphological and reproductive characteristics. *Journal of Insect Physiology*, 57:514–520

Manchanda SK, Sachan GC, Rathore YS (1980) Effects of hosts on the morphometrics and phase status of *Schistocerca gregaria* Forskal. *Journal of Applied Entomology*, 89:26–31

McAdam, A.G., Garant, D., Wilson, A.J. (2014). The effects of other' genes: maternal and other indirect genetic effects. In: Quantitative genetics in the wild. Oxford University Press, pp 84–103

McCaffery AR, Simpson SJ, Islam MS, Roessingh P (1998) A gregarizing factor present in the egg pod foam of the desert locust *Schistocerca gregaria*. *Journal of Experimental Biology*, 201:347–363

Nickerson B (1956) Pigmentation of hoppers of the desert locust (*Schistocerca gregaria* Forsk) in relation to phase coloration. *Anti-Locust Bulletin*, 24:1–34

Nolte DJ (1962) Strain differentiation in locusts. In: *Proceedings of the Second Congress of the South African Genetics Society*. pp 96–100

Nolte DJ (1965) The pigmentation of locusts. *South African Journal of Science*, 61:173–178

Osborne JW, Carolina N (2010) Improving your data transformations : Applying the Box-Cox transformation. *Practical Assessment, Research and Evaluation*, 15:1–9

Papillon M (1968a) Facteurs écologiques et phases chez le criquet pèlerin, *Schistocerca gregaria* (Forsk.). II-Influence de la densité des populations. *Bulletin biologique de la France et de la Belgique*, 102:271–307

Papillon M (1968b) Facteurs écologiques et phases chez le criquet pèlerin, *Schistocerca gregaria* (Forsk.). I-Influence de la photopériode et de la température. *Bulletin biologique de la France et de la Belgique*, 102:85–139

Pener MP, Simpson,SJ (2009). Locust phase polyphenism: an update. *Advances in Insect Physiology*, 36, 1–272

Pélissié B, Piou C, Jourdan-Pineau H, Pagès C, Blondin L, Chapuis MP (2016). Extra molting and selection on nymphal growth in the desert locust. *PLoS One*, 11, 1–18

Roffey J, Magor JI (2003). Desert locust population dynamics parameters. *Technical Series-Desert Locust Field Research Stations* (FAO)

Simpson SJ, Miller GA (2007) Maternal effects on phase characteristics in the desert locust, *Schistocerca gregaria*: A review of current understanding. *Journal of Insect Physiology*, 53: 869-876

Stower WJ, Davies DE, Jones IB (1960) Morphometric studies of the desert locust, *Schistocerca gregaria* (Forsk.). *Journal of Animal Ecology*, 29:309–339

Tanaka S, Maeno K (2006). Phase-related body-color polyphenism in hatchlings of the desert locust, *Schistocerca gregaria*: re-examination of the maternal and crowding effects. *Journal of Insect Physiology*, 52: 1054–106

Van Huis A, Woldewahid G, Toleubayev K, Van Der Werf W (2008) Relationships between food quality and fitness in the desert locust, *Schistocerca gregaria*, and its distribution over habitats on the Red Sea coastal plain of Sudan. *Entomologia Experimentalis et Applicata*, 127:144–156

Ward D (2009). The biology of deserts. Oxford: Oxford University Press

Wardhaugh K, Ashour Y, Ibrahim AO, Khan, AM, Bassonbol, M (1969) Experiments on the incubation and hopper development periods of the desert locust (*Schistocerca gregaria* Forskål) in Saudi Arabia. *Anti-Locust Bulletin*, 45:9–14
